# Peripheral-blood gene expression profiling studies for coronary artery disease and its severity in Xinjiang population in China

**DOI:** 10.1186/s12944-018-0798-1

**Published:** 2018-07-18

**Authors:** Meng Liu, Shubin Jiang, Yu Ma, Jun Ma, Waseem Hassan, Jing Shang

**Affiliations:** 1 State Key Laboratory of Natural Medicines, China Pharmaceutical University, Nanjing, 210009 Jiangsu China; 2 Jiangsu Key Laboratory of TCM Evaluation and Translational Research, China Pharmaceutical University, Nanjing, 211198 Jiangsu China; 3 Cancer Prevention and Research Institute, The affiliated Tumor Hospital of Xinjiang Medical University, Urumqi, 830011 Xinjiang China; 4 Department of Coronary Care Unit, Traditional Chinese Medicine Hospital Affiliated to Xinjiang Medical University, Urumqi, 830000 Xinjiang China; 5 Department of Clinical Laboratory, The Fourth People’ Hospital of Urumqi, Urumqi, 830002 Xinjiang China; 6 Department of Pharmacy, COMSATS University Islamabad, Lahore Campus, Lahore, Pakistan

**Keywords:** Coronary artery disease, Coronary stenosis, Microarray analysis, Genetic variation, Toll-like receptor signaling pathway

## Abstract

**Background:**

Alterations in gene expression in peripheral blood cells play a **curtail** role in the presence and extent of coronary artery disease (CAD), but its severity reflected by gene expression alterations in peripheral blood cells is still unknown in Xinjiang population in China.

**Methods:**

Global gene expression profiling in peripheral blood was used to explore differentially expressed genes in coronary artery stenosis patients. RNA was extracted from peripheral blood of 9 controls without coronary stenosis and 21 cases with angiographically CAD. The extent of CAD severity was categorized angiographically as no CAD, mild CAD (20 to 50% luminal diameter stenosis [LDS]), moderate CAD (50 to 75% LDS) and severe CAD (≥75% LDS). Differentially expressed genes related with CAD severity from peripheral blood cells were screened by linear mixed effects analysis using the lme4 package in R. Then the differentially expressed genes that gradually up-regulated or down-regulated were enriched by Gene Ontology (GO) functional annotation and Kyoto Encyclopedia of Genes and Genomes (KEGG) pathway analysis.

**Results:**

The most significantly enrichments were toll-like receptor signaling pathway, immune responses, translational processes, cellular growth, inflammation and metabolic processes. Combined with NCBI-GeneRIF and PubMed analysis, we focused on the 12 genes associated with toll-like receptor signaling pathway in the extent of coronary artery stenosis patients. Receiver operating characteristic (ROC) analysis of 12 genes associated with toll-receptor signaling pathway in the 236 CAD patients from GEO database demonstrated that 12 genes expression could predict severe CAD with an area under the curve of 0.67, sensitivity of 77.65% and specificity of 51.52%.

**Conclusion:**

These results suggest that 12 genes associated with toll-like receptor signaling pathway in peripheral-blood cells reflect the presence and extent of CAD severity in Xinjiang population in China.

**Electronic supplementary material:**

The online version of this article (10.1186/s12944-018-0798-1) contains supplementary material, which is available to authorized users.

## Background

The epidemiological study showed that coronary artery disease (CAD) and coronary atherosclerosis are the largest source of morbidity and mortality in the general population worldwide [1, 2]. The development of cardiovascular disease (CVD) is a multistep process from normal vessel to severe stenosis, accompanied by the molecular alterations. The aberrant molecular alterations and environmental modifications which give rise to cardiovascular disease occur before morphological abnormality of the tissue [3]. Hence, molecular alterations study can provide the valuable information in early detection of CAD severity in asymptomatic patients. Besides, genes expression variants in peripheral blood cells associated with the severity extent of CAD have also been reported [4]. Therefore, investigation of gene expression variation in extent of CAD severity could become a powerful way to understand the causes of CAD [5].

Recent studies have demonstrated that microarray analysis of peripheral blood cells is a practical way to investigate gene expression variations, which not only hint genetic predisposition but also reflect the activity of disease, environmental modifier effects, and treatment responses [6, 7]. A recent report has suggested the importance of gene expression variations in peripheral blood by investigating the pathophysiology of vascular calcification between African Americans and Whites [8]. Previous reports have also mentioned that gene expression profiling of peripheral blood is correlated with the severity of CAD and may be a strong predictor of cardiovascular outcomes [9, 10].

In the present research, we performed the microarray analysis method to investigate the gene expression profiling of whole blood in the extent of CAD severity in Xinjiang population of China. Differentially expressed genes related with the extent of CAD severity were identified by linear mixed effects analysis. Afterwards, differentially expressed genes that gradually up-regulated or down-regulated were enriched by GO functional annotation and KEGG pathway analysis. Finally, we found for the first time that 12 genes associated with toll-like receptor signaling pathway were related with CAD severity. Moreover 12 genes were validated in 236 CAD samples from GEO dataset by ROC analysis. Consequently, identifying the whole human transcriptome of peripheral blood in CAD patients could provide new molecular mechanisms of the development of atherosclerosis in the extent of CAD patients in Xinjiang population in China.

## Results

### Global gene expression profile analysis of CAD patients

**Fig. 1:**
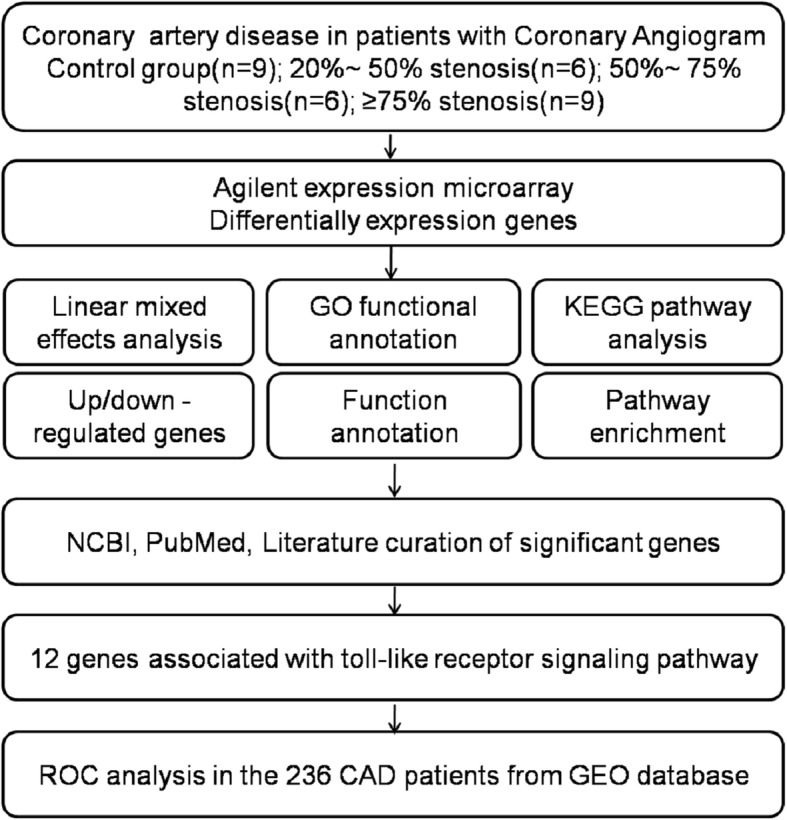
Schematic overview of the workflow. Peripheral blood were obtained from 9 control groups and 21 CAD patients who underwent angiography. In the study, controls were defined by coronary angiography as 0% stenosis and mild CAD as 25% ~ 50% stenosis, moderate CAD as 50% ~ 70% stenosis, severe CAD as ≥75% stenosis

To identify differential genes expressed in peripheral blood that may be associated with the extent of coronary artery stenosis, we performed a multistep method, starting with genes discovery from microarrays and validated followed by ROC analysis in CAD samples from GEO dataset (GSE10195). An overview of the overall study design is shown in Fig. 1. The clinical characteristics of the CAD patients in Xinjiang population in China are shown in Table 1. CAD patients had higher cholesterol levels than controls, and hypertension was more frequent.

**Table 1:** Clinical characteristics of control group and the extent of coronary artery disease patients

	Control group (*N* = 9)	Mild stenosis group (*N* = 6)	Moderate stenosis group (*N* = 6)	Severe stenosis group (*N* = 9)	*P* ^Value^
Age ± sd (years)	43.66 ± 5.61	50.36 ± 4.31	52.66 ± 5.19	50.16 ± 5.36	0.772
BMI ± sd (kg/m^2^)	23.20 ± 2.52	25.20 ± 2.52	26.50 ± 1.92	28.13 ± 3.36	0.640
Hx of Hyperlipidemia (*n*, %)	0	2 (33%)	4 (66%)	6 (66%)	0.541
Hx of Hypertension (*n*, %)	0	2 (33%)	3 (50%)	4 (44%)	0.813
Hx of Diabetes (*n*, %)	0	0	0	0	
Hx of Smoking (*n*, %)	2 (22%)	3 (50%)	3 (50%)	8 (88%)	0.750
Coronary arterial stenosis (*n*, %)	0	39 ± 4%	67 ± 5%	81 ± 6%	<0.001
LV ejection fraction (%)	50.7 ± 1%	55.76 ± 2%	59.16 ± 2%	62.1 ± 4%	0.780
Biometrics					
Systolic blood pressure ± sd (mmHg)	118.88 ± 6.00	120.38 ± 9.39	122.33 ± 6.00	138.4 ± 8.93	0.813
Diastolic blood pressure ± sd (mmHg)	75.55 ± 7.26	71 ± 3.22	77.44 ± 8.09	87.9 ± 4.22	0.731
Laboratory parameterst					
Total cholesterol ± sd (mmol/L)	3.67 ± 0.34	3.77 ± 0.45	3.99 ± 0.34	4.2 ± 1.19	0.042
LDL-cholesterol ± sd (mmol/L)	2.19 ± 0.38	2.91 ± 0.58	2.98 ± 0.71	3.25 ± 0.77	0.087
HDL-cholesterol ± sd (mmol/L)	1.94 ± 0.91	1.11 ± 0.25	1.24 ± 0.21	1.09 ± 0.45	0.083
Triglycerides (mmol/L)	1.03 ± 0.45	1.64 ± 0.45	2.64 ± 0.45	3.07 ± 0.85	0.032
Apolipoprotein A (g/L)	1.16 ± 0.06	1.61 ± 0.06	1.96 ± 0.06	2.15 ± 0.31	1
Apolipoprotein B (g/L)	0.76 ± 0.06	1.09 ± 0.06	1.29 ± 0.08	1.99 ± 0.25	1
Hb_AIC_ (*%*)	4.45 ± 0.46	5.65 ± 0.36	5.97 ± 0.46	6.01 ± 0.87	0.431
Hs-CRP (mg/L)	4.34 ± 0.14	3.506 ± 0.14	3.906 ± 0.14	4.106 ± 1.00	0.866
Hematocrit (HCT)	4.13 ± 0.23	4.31 ± 0.13	4.33 ± 0.32	4.79 ± 0.15	0.753
Creatinine (mg/dL) (*%*)	73.13 ± 11.17	76.13 ± 10.17	78.13 ± 13.17	76.34 ± 12.25	0.201
White blood cell count (10^9^/L)	6.12 ± 1.12	6.92 ± 1.21	7.12 ± 1.32	8.02 ± 2.26	0.567
Medications					
Statins (*n*, %)	0	3 (50%)	4 (66%)	7 (77%)	0.850

**Fig. 2:**
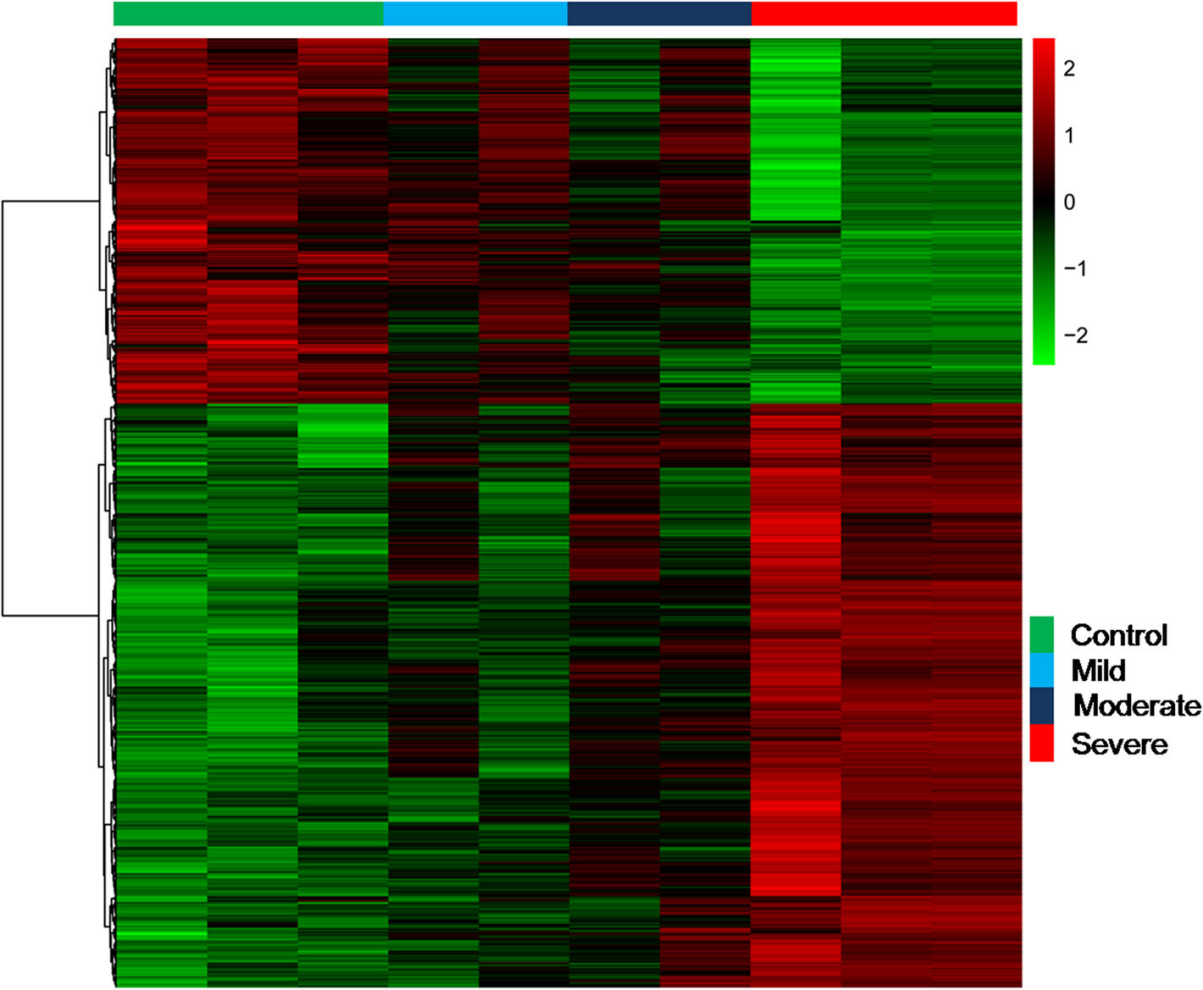
Hierarchical cluster analysis of differentially expressed genes. Differentially expressed genes as identified by the Gene spring software are represented in rows, and the different samples are represented in columns. Red indicates relative overexpression while green represent relative underexpression level, Black values represent central expression values

Hierarchical clustering of the data was used to observe the overall gene expression difference in the samples. All CAD samples were clustered together and separated from the normal samples. It was observed that different extent of coronary stenosis samples have different expression values in Fig. 2. Compared with controls, a total of 422 genes showed significantly differential expressions (*p* < 0.05 and fold change>2.0) with 278 up-regulated genes and 144 down-regulated genes.

**Fig. 3:**
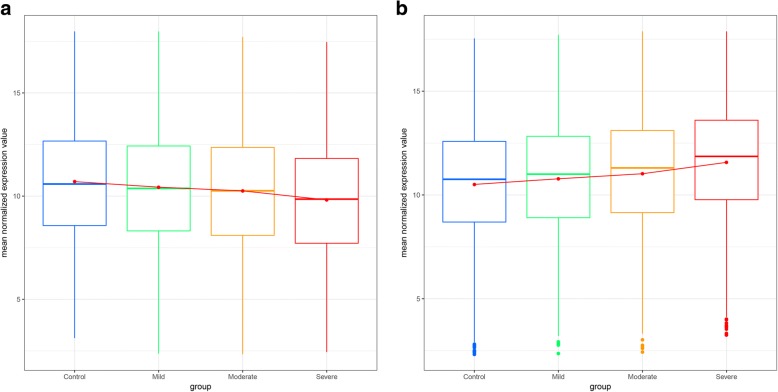
Genes expression patterns in the differential coronary artery stenosis patients. **a** gradually downregulated genes from control group to severe coronary artery stenosis group. **b** gradually upregulated genes from control group to severe coronary artery stenosis group

Then, a linear mixed effects analysis was used to identify the coronary artery stenosis-related differentially expressed genes using a false discovery rate (FDR) ≤0.05, which yielded 1328 genes, of which 817 were gradually up-regulated (Additional file 1: Table S1), and 511 were down-regulated (Additional file 2: Table S2) from controls to severity coronary artery stenosis groups in the Fig. 3. These coronary artery stenosis-related differentially expressed genes were sensitive to the extent of coronary artery stenosis.

### GO and KEGG analysis of the coronary stenosis-related differentially expressed genes

**Fig. 4:**
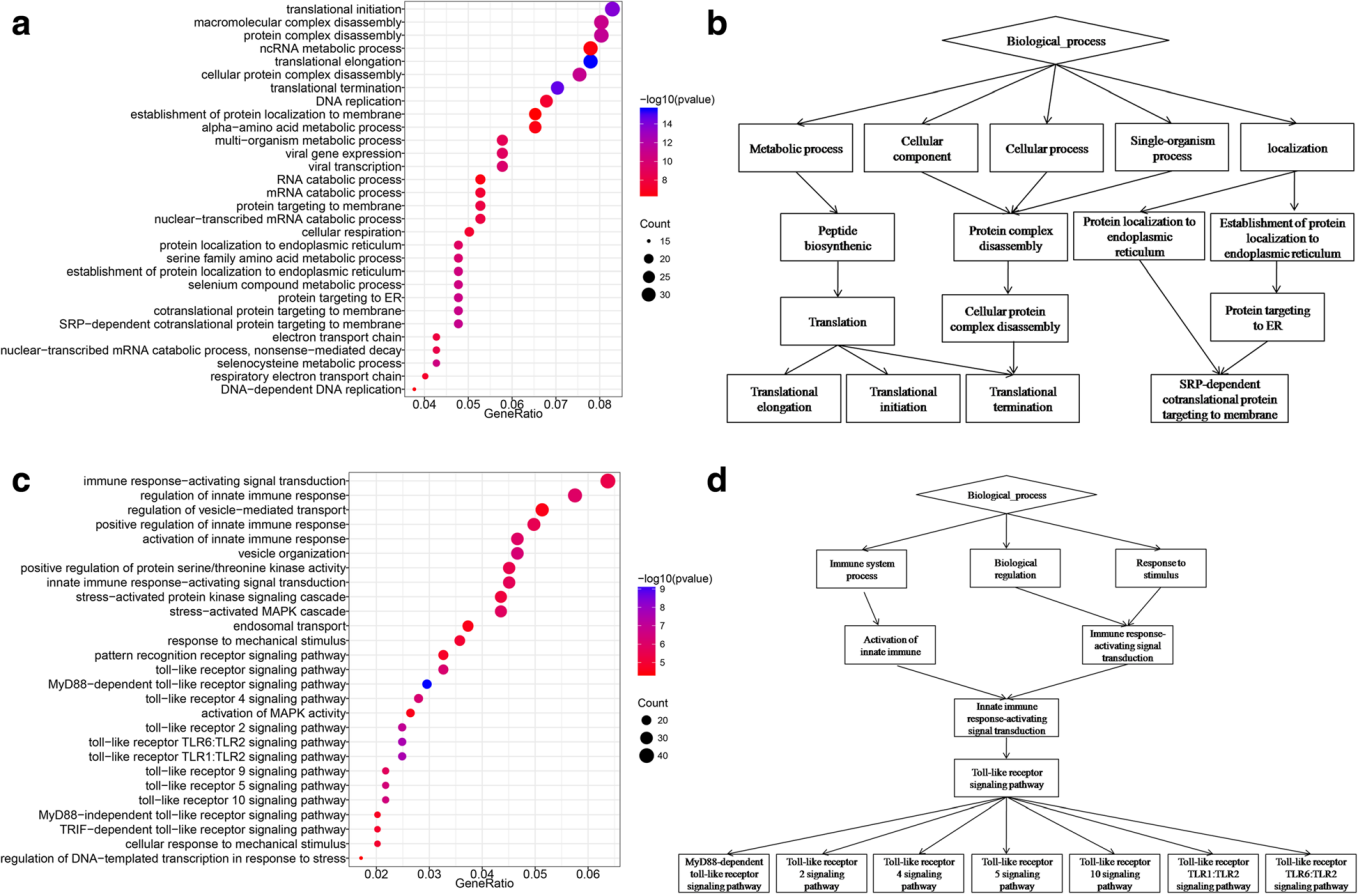
Functional annotation of differentially expressed genes. **a** Enrichment analysis for the same gene ontology categories in GO terms derived from downregulated genes. **b** GO functional networks of downregulated genes, Color of node represented significantly GO terms. **c** Enrichment analysis for the same gene ontology categories in GO terms derived from upregulated genes. **d** GO Functional networks of upregulated genes, Color of node represented significantly GO terms

The functions of 817 gradually up-regulated and 511 gradually down-regulated genes were examined by GO enrichment and KEGG analysis. Size of node represented the numbers of genes and color of node indicated the *p*-value of genes in the GO terms. The *p*-value of each GO term reflects the enrichment in frequency of GO term. The GO functional enrichment of gradually up-regulated genes showed that immune responses, toll-like receptor signaling pathway and MAPK were related with the extent of CAD. GO terms associated with gradually down-regulated genes were enriched for several terms including translational initiation, translational elongation, translational termination and SRP-dependent cotranslational protein targeting to membrane. Results of the GO annotation based enrichment analysis of gradually up-regulated and down-regulated genes were shown in Fig. 4. Yellow circle represented the name of enrichment KEGG pathway and size of yellow circle indicated the *p*-value of KEGG pathway, the node around yellow circle suggested genes that enriched KEGG pathway.

**Fig. 5:**
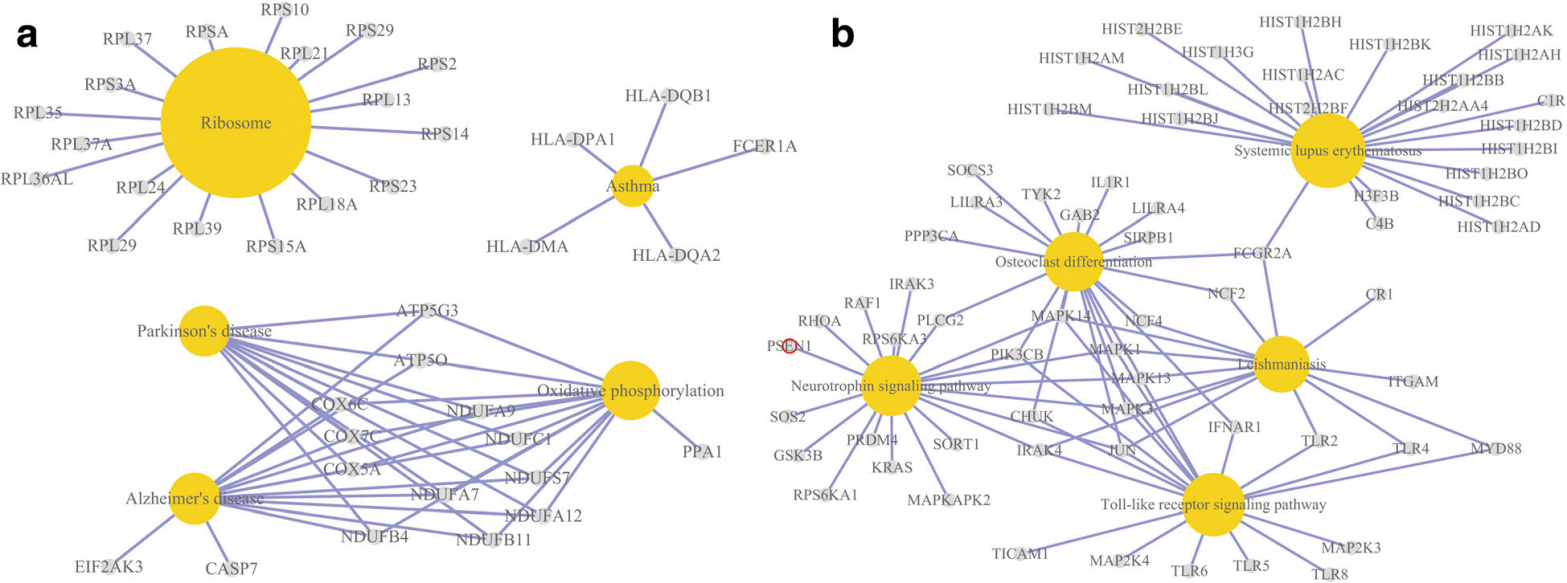
KEGG pathway enrichment analysis results. **a** KEGG pathways mediated by gradually downregulated genes. **b** KEGG Pathways mediated by gradually upregulated genes

KEGG enrichment analysis revealed that five pathways were related with up-regulated genes and five pathways were significant in down-regulated genes (*p* < 0.05) (Fig. 5). These results indicated that KEGG pathways mediate immune system, metabolic diseases and oxidative phosphorylation.

These significantly enriched pathways demonstrated that toll-like receptor signaling pathway occurred in multiple signaling and cellular mechanisms. Twelve direct interactions genes with toll-like receptor signaling pathway were picked out from multiple network in up-regulated KEGG pathways, while the down-regulated genes did not interact with toll-like receptor signaling pathway (Table 2).

**Table 2:** 12 differentially expressed genes associated with toll-like receptor signaling pathway in the extent of coronary artery stenosis patients

Gene symbol	Gene description	Trend 1	Trend 2	Trend 3	*p*-value
MAPK3	Mitogen-activated protein kinase 3	0.1681	0.3593	0.9498	0.00045
MAPK1	Mitogen-activated protein kinase 1	0.2921	0.5725	1.1005	0.00196
CHUK	Conserved helix-loop-helix ubiquitous kinase	0.1854	0.2862	0.6197	0.00622
PIK3CB	Phosphatidylinositol-4,5-bisphosphate 3-kinase catalytic subunit beta	0.4401	0.4743	1.1232	0.00830
MAPK14	Mitogen-activated protein kinase 14	0.3399	0.7724	1.9161	0.00960
TLR4	Toll like receptor 4	0.6287	0.8780	1.9592	0.01065
IFNAR1	Interferon alpha and beta receptor subunit 1	0.3049	0.9418	1.0834	0.01283
MAPK13	Mitogen-activated protein kinase 13	0.1807	0.3373	0.7193	0.02156
JUN	Jun proto-oncogene	0.3740	0.4746	1.0779	0.02579
TLR2	Toll like receptor 2	0.2299	0.4885	1.2470	0.03053
MYD88	Myeloid differentiation primary response 88	0.4350	0.6164	0.8260	0.03805
IRAK4	Interleukin 1 receptor associated kinase 4	0.0101	0.3213	0.7935	0.04165

Trend 1: Mid CAD compared with control group; Trend 2: Moderate CAD compared with mid group; Trend 3: Severe CAD compared with moderate group

In the end, integrated bioinformatics pipeline associated with literature curation, GeneRIF and publications linked under “Related Articles” in PubMed indicated that 12 genes related with toll-like receptor signaling pathway were considered for the further analysis.

### ROC analysis

**Fig. 6:**
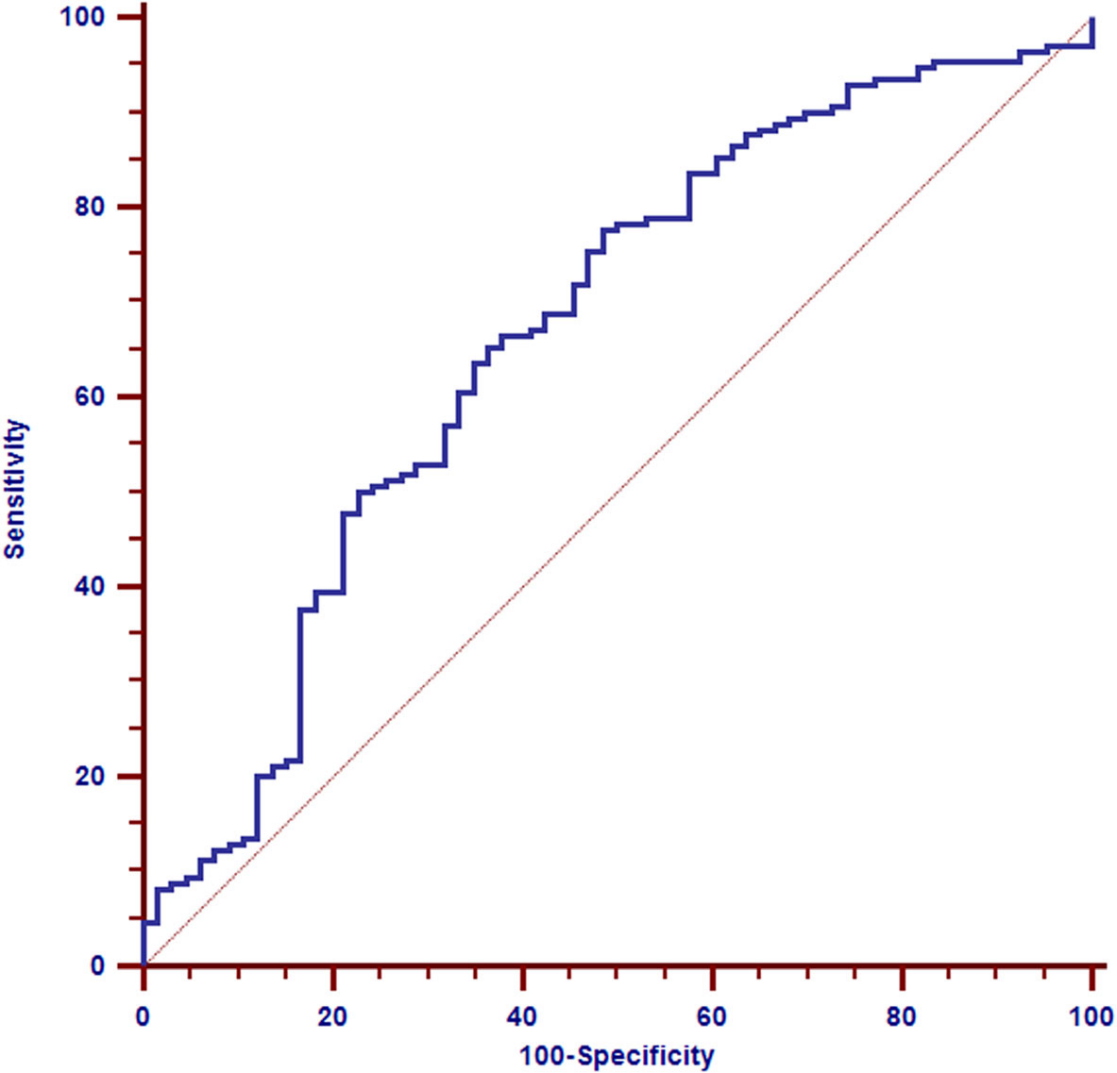
ROC analysis. Twelve genes associated with toll-like receptor signaling pathway were validated by ROC analysis in 236 CAD patients from GEO dataset. The AUC was 0.67 ± 0.037, and sensitivity was 77.65% and specificity was 51.52%

ROC analysis demonstrated that 12 genes validated in set of 236 CAD patients (170 cases, 66 controls) from GEO dataset (GSE10195) yielded an area under the curve (AUC) of 0.67 (95% CI = 0.71 to 0.80), sensitivity 77.65% and specificity 51.52% (Fig. 6).

## Discussions

We used the microarray methods in current study to investigate differences in overall gene expression profiles in peripheral blood obtained from different extent of coronary artery stenosis patients in Xinjiang population of China. We identified that differentially expressed genes with gradient coronary artery stenosis-related expression trends from normal group to CAD severity patients by linear mixed effects analysis. Gradually up-regulated or down-regulated genes were enriched by GO functional annotation and KEGG pathway analysis. Combined with “Related Articles” in PubMed, 12 genes associated with toll-like receptor signaling pathway were identified and validated in 236 CAD samples from GEO dataset (GSE10195) that AUC was 0.67 (95% CI 0.71 to 0.80). In addition, the expression of 12 genes associated with the TLR signaling pathway was not correlated with the circulating levels of triglycerides and cholesterol in control and patient groups. Triglycerides and cholesterol levels were balanced in control and patients, because they were not significant difference. These results suggesting that 12 genes related with toll-like receptor signaling pathway are the significant CAD predictor.

Extent of coronary artery stenosis is often used to investigate the development of CAD. James A. Wingrove et al. identified transcript expression in peripheral blood, which was associated with extent of coronary artery stenosis patients [11]. Sharon Cresci et al. found that peroxisome proliferator-activated receptor pathway gene polymorphism was associated with extent of coronary artery disease in patients with type 2 diabetes [12]. Predictability of coronary artery disease progression is of vital importance for clinical decisions like revascularization strategies. Therefore, these models were designed to evaluate the transcriptome in the presence of coronary artery disease or to predict the extent of disease from the transcriptome. Through linear mixed effects analysis statistical model building, gradually up-regulated and down-regulated genes were found. Furthermore, a marker set consisting of 12 genes were identified by GO and KEGG enrichment analysis. Moreover, ROC analysis from CAD patients in GEO dataset showed that accurate prediction of CAD progression.

Enrichment analysis of GO terms and KEGG pathways related with gradually up-regulated and down-regulated genes provide several known and novel molecular mechanisms associated with coronary artery stenosis. Our results demonstrated that inflammatory genes (MAPK1, MAPK3, MAPK13, MAPK14, JUN) are significantly up-regulated in the CAD patients. Guo Nan et al. reported that mitogen-activated protein kinase-1(MAPK1) and its downstream factors of hypoxia-inducible factor-1 (HIF-1) and heme oxygenase-1 (HO-1) were involved in regulating development of coronary artery disease [13]. Jiny Nair et al. employed network analysis to identify that JUN, nuclear factor kappa B1(NF-κB) and signal transducer and activator of transcription 3 (STAT3) were three core transcription factors for CAD [14]. Previous study demonstrated that MAPK13 encodes a p38δ MAP kinase protein and is activated by pro-inflammatory cytokines and cellular stress [15]. Moreover, inhibition of a key signaling molecule of the p38α MAPK/MAPK14 might prevent inflammatory cascades in atherosclerosis [16]. Toll-like receptors (TLRs) involved in innate immunity and pathogen recognition have been identified to play a vital role in the progression of CAD. In this study, we have found that TLR2 and TLR4 were significantly up-regulated in CAD patients. Multiple studies have demonstrated that TLR2 and TLR4 were up-regulated in atherosclerotic plaques and peripheral blood cells associated with severe atherosclerotic disease [17–19]. Dandan Sun et al. suggested that toll-like receptor 4 (TLR4) rs11536889 polymorphism was a genetic factor in the development and extent and severity of CAD [20]. Liang Shao et al. showed that TLR4 mRNA and myeloid differentiation primary response 88 (MyD88) were significantly increased in patients with coronary artery stenosis [21, 22]. Additionally, the expression of MyD88 in peripheral blood cells of CAD patients was up-regulated in our study. Altogether, these results suggested that inflammation and innate immunity system were activated in the initiation and progression of CAD.

Apart from these known genes, several genes (IFNAR1, IRAK4, CHUK and PIK3CB) not previously associated with CAD were identified to be differentially expressed in extent of CAD patients. Kristen Lynette Hosey et al. suggested that IFNAR1-mediated signaling play a critical role in IFN-α gene transcription [23]. Alexander asmussen et al. reported that the interleukin-1 receptor-associated kinase (IRAK)4 was the main kinase to further propagate TLR signaling [24]. Besides, previous studies shown that conserved helix-loop-helix ubiquitous kinase (CHUK), IRAK3 and MYD88 were encoding proteins with NF-κB activating or inhibiting properties [25]. PI3K, an intracellular enzyme, is involved in many biological processes including cell growth, differentiation, survival and motility [26]. Collectively, GO functional enrichment analysis showed that up-regulated 12 genes were implicated in toll-like receptor signaling pathway. Similar to GO functional enrichment, toll-like receptor signal transduction events were also observed in the KEGG pathway analysis. Altogether, bioinformatics algorithms (linear mixed effects analysis) was firstly used to screen differentially expressed genes in the different extent of coronary artery disease. And then these differentially expressed genes were enriched toll-like receptor signaling pathway by GO functional annotation and KEGG pathway analysis. These results showed that 12 genes associated with toll-like receptor signaling pathway played a vital role in the development of coronary artery stenosis. It has been elucidated that toll-like receptor signaling pathway promoted the secretion of adhesion molecules and chemokines, the proliferation and migration of VSMCs, as well as the infiltration of monocyte/macrophage and formation of foam cells [27]. Therefore these studies demonstrated that toll-like receptor signaling pathway participated in development stage of coronary artery stenosis. Further study of toll-like receptor signaling pathway of differentially regulated transcription factors and their downstream target genes could provide additional insights into the molecular mechanism of CAD.

Conversely, GO functional annotation revealed that down-regulation of several genes was the process of transcription and translation, many of them involved in the regulation of translational initiation, elongation and termination. Furthermore alpha-amino acid metabolic process and multi-organism metabolic process in GO enrichment analysis were also down-regulated. KEGG pathway analysis demonstrated that down-regulated genes were related to chronic disease such as parkinson disease and alzheimer disease. Additionally, we observed that oxidative phosphorylation genes enriched in KEGG pathway were significantly down-regulated in the extent of CAD patients. Thus, these results reflected that the gradually down-regulated genes are correlated with chronic diseases such as cardiovascular disease and neurodegenerative disorders as well as metabolic abnormalities. These results might be explained by the fact that the patients were on statins (**atorvastatin**) and had acceptable risk factor control of chronic diseases [28], which would significantly affect gene expression in peripheral blood.

Our findings had several potential limitations. First, the samples number in our study was limited, and the samples used in validation set should be enlarged. Furthermore, although 12 genes associated with toll-like receptor signaling pathway could predict the progression of CAD, the molecular mechanism of toll-like receptor signaling pathway related 12 genes in CAD progression was still unknown, and further study should be performed in vitro and in vivo to clarify the mechanism. Finally, further network analysis should be performed to identify key hub node genes and core transcriptional regulators in the extent of CAD patients.

## Conclusions

In conclusion, our study identified 12 genes associated with toll-like receptor signaling pathway were significantly associated with extent of CAD patients in Xinjiang population of China. Moreover, our results suggested that variation of 12 genes expression in peripheral blood might impact on vascular biology and provide valuable information about CAD progression. To the best of our knowledge, this is the first report of a significant association of toll-like receptor signaling pathway genes with extent of the coronary artery stenosis. Future research should focus on large enough samples to be able to evaluate genetic effects in the extent of CAD patients in Xinjiang population in China.

## Methods

### Collection of clinical samples

The CAD peripheral blood samples were collected from 9 healthy volunteers and 21 CAD patients who underwent angiography at the affiliated hospital of traditional Chinese medicine Xinjiang medical university from 2014 to 2016. All patients provided informed, written consent to use their samples for this study and consent for publication. Manuscripts reporting studies involving human participants, human data or human tissue were approved by the ethics committee of affiliated hospital of traditional Chinese medicine Xinjiang medical university in china (GZR201401-10). The clinicopathological information was obtained from all patients. Clinical inclusion and exclusion criteria were described as previous study [29]. Clinical indication for coronary angiography, CAD severity was defined angiographically luminal diameter obstruction in at least one major coronary artery vessel as follows: no CAD, mid CAD (20–50% luminal diameter stenosis [LDS]), moderate CAD (50–75% LDS) and severe CAD (≥75% LDS). Patients were excluded from the study for the following reasons: pregnancy; acute infection; moderate or greater severity of congestive heart failure (New York Heart Association class III or IV); left ventricular ejection fraction <0.35; moderate or severe heart failure; myocardial infarction; cardiogenic shock; stroke; coronary artery bypass surgery; severe valvular heart disease; presence of visual coronary collaterals; cardiac transplantation; uncontrolled hypertension (defined as either a resting diastolic blood pressure of ≥100 mmHg or a resting systolic blood pressure of ≥180 mmHg); triglyceride (TG) level ≥ 400 mg/dL; hematologic, neoplastic, metabolic, gastrointestinal or endocrine dysfunction; uncontrolled diabetes (defined as glycosylated hemoglobin >10%); active liver disease or hepatic dysfunction, as determined by aspartate aminotransferase (AST [SGOT]), alanine aminotransferase (ALT [SGPT]) or bilirubin levels ≥1.5 × ULN; secondary causes of hyperlipoproteinemia, such as uncontrolled primary hypothyroidism (defined as thyroid stimulating hormone [TSH] ≥1.5 × ULN), nephrotic syndrome, and/or renal dysfunction (serum creatinine ≥2.0 mg/dL [177 μmol/L]); renal insufficiency (creatinine > 2.0 mg/dL or BUN > 40 mg/dL).

### Microarray analysis methods

Total RNA was extracted from peripheral blood cells in nine healthy volunteers and 21 CAD patients using the RNeasy kit (Qiagen, USA), and then three RNA samples in each group were pooled for microarray analysis. The RNA concentrations were measured by an ND-1000 UV-VIS spectrophotometer (NanoDrop Technologies, USA). RNA integrity was quantified by agilent 2100 bioanalyzer (Agilent, USA) and RNA with integrity number (RIN) ≥ 6.5 was used in microarray analysis. All samples were tested by a whole human genome oligo microarray (4 × 44 K, Agilent Technologies) according to the manufacturer’s protocol. The genes raw data (median values > 100) were normalized by the median scale method using the R package “limma” [30]. An expression matrix of 32,797 genes was used for the subsequent study.

R and R package “lme4” were used to do a linear mixed effects analysis of the relationship between gene expression and the extent of CAD stenosis. Genes with a false discovery rate (FDR) ≤ 0.05 were considered significant.

### Functional annotation and KEGG analysis

GO functional enrichment analysis was performed in terms of biological processes, cellular components and molecular functions. Moreover, biological pathways mediated by differentially expressed genes were also defined by KEGG from annotation, visualization and integrated discovery. CAD severity-related gradually up-regulated and down-regulated genes were analyzed by GO functional enrichment and KEGG analysis. Finally, the close interacted differentially expressed genes in GO and pathway networks were identified based on previous study, and combined with scientific literature related articles by pubmed analysis.

### ROC analysis

The 236 CAD samples from GEO database (GSE10195) were used and validated by the receiver operator curve (ROC) analysis. The 12 genes associated with the toll-like receptor signaling pathway were calculated AUC, sensitivity and specificity.

### Statistical analysis

All statistical tests were two-sided, and a 5% level of significance was used. The statistical analyses were performed using R software (http://www.r-project.org). R and linear model was used to identify the gradually down-regulated or up-regulated genes from the no CAD to mild CAD, moderate CAD and severe CAD. R and R package “clusterProfiler” and “GeneAnswers” and “KEGG.db” were used to do GO and KEGG enrichment analysis between gene expression and the extent of CAD stenosis. ROC analyses were performed with MedCalc Software (version 13.1.2, Medcalc Statistical Software, Ostend, Belgium).

## Additional files

Additional file 1: **Table S1.** Gene list of the coronary artery stenosis-related up-regulated genes identified by linear mixed effects model analysis. (XLSX 77 kb)

Additional file 2: **Table S2.** Gene list of the coronary artery stenosis-related down-regulated genes identified by linear mixed effects model analysis. (XLSX 52 kb)

## Abbreviations

CAD: Coronary artery disease
CVD: Cardiovascular disease
FDR: False discovery rate
GO: Gene Ontology
KEGG: Kyoto Encyclopedia of Genes and Genomes
LDS: Luminal diameter stenosis
ROC: Receiver operating characteristic
TLR: Toll-like receptor
